# A Novel Dietary Assessment Method to Measure a Healthy and Sustainable Diet Using the Mobile Food Record: Protocol and Methodology

**DOI:** 10.3390/nu7075226

**Published:** 2015-07-03

**Authors:** Amelia J. Harray, Carol J. Boushey, Christina M. Pollard, Edward J. Delp, Ziad Ahmad, Satvinder S. Dhaliwal, Syed Aqif Mukhtar, Deborah A. Kerr

**Affiliations:** 1 School of Public Health, Curtin University, GPO Box U1987, Perth 6845, Australia; E-Mails: C.Pollard@curtin.edu.au (C.M.P.); S.Dhaliwal@curtin.edu.au (S.S.D.); D.Kerr@curtin.edu.au (D.A.K.); 2 Epidemiology Program, University of Hawaii Cancer Center, Honolulu, HI 96813, USA; E-Mail: CJBoushey@cc.hawaii.edu; 3 Video and Image Processing Laboratory, School of Electrical and Computer Engineering, Purdue University, West Lafayette, IN 47907-2035, USA; E-Mails: ace@ecn.purdue.edu (E.J.D.); zahmad@purdue.edu (Z.A.); 4 Department of Health Western Australia, Perth 6004, Australia; 5 Centre for Population Health Research, Curtin University, GPO Box U1987, Perth 6845, Australia; E-Mail: Aqif.Mukhtar@curtin.edu.au

**Keywords:** sustainable diet, healthy diet, dietary assessment, mobile food record, technology

## Abstract

The world-wide rise in obesity parallels growing concerns of global warming and depleting natural resources. These issues are often considered separately but there may be considerable benefit to raising awareness of the impact of dietary behaviours and practices on the food supply. Australians have diets inconsistent with recommendations, typically low in fruit and vegetables and high in energy-dense nutrient-poor foods and beverages (EDNP). These EDNP foods are often highly processed and packaged, negatively influencing both health and the environment. This paper describes a proposed dietary assessment method to measure healthy and sustainable dietary behaviours using 4-days of food and beverage images from the mobile food record (mFR) application. The mFR images will be assessed for serves of fruit and vegetables (including seasonality), dairy, eggs and red meat, poultry and fish, ultra-processed EDNP foods, individually packaged foods, and plate waste. A prediction model for a *Healthy and Sustainable Diet Index* will be developed and tested for validity and reliability. The use of the mFR to assess adherence to a healthy and sustainable diet is a novel and innovative approach to dietary assessment and will have application in population monitoring, guiding intervention development, educating consumers, health professionals and policy makers, and influencing dietary recommendations.

## 1. Introduction

Recent evidence would suggest that eating a diet that increases environmental sustainability has the potential to also benefit health [1,2,3,4]. Worldwide overweight and obesity rates are rising, posing significant costs at an individual and societal level [5]. In Australia, the direct cost spent annually on overweight and obesity is estimated to be at least $21 billion [6]. The overconsumption of kilojoules above an individual’s energy requirements (resulting in weight gain) is environmentally unsustainable and places burden on the future food supply [7]. Hence, there may be considerable health and environmental benefits in assessing the impact of dietary behaviours and practices on the food supply [8,9]. Research on the effects of diet on the environment is rapidly emerging, particularly the area of life cycle assessment- a method for measuring the carbon footprint (amount of greenhouse gas emissions) of food products throughout production [10,11]. However, identifying a healthy and sustainable diet that meets the nutrient requirements of all populations groups and cultures is complex and challenging [1,8,12,13]. Researchers have identified the need to identify dietary patterns that provide adequate nutrition at a low environmental cost [14], but methods to do so have focused on the assessment of typical diets and food choices at a population level [2,13] rather than individual dietary behaviours. Therefore, there is limited evidence on whether current individual dietary patterns align with a sustainable diet.

**Figure 1:**
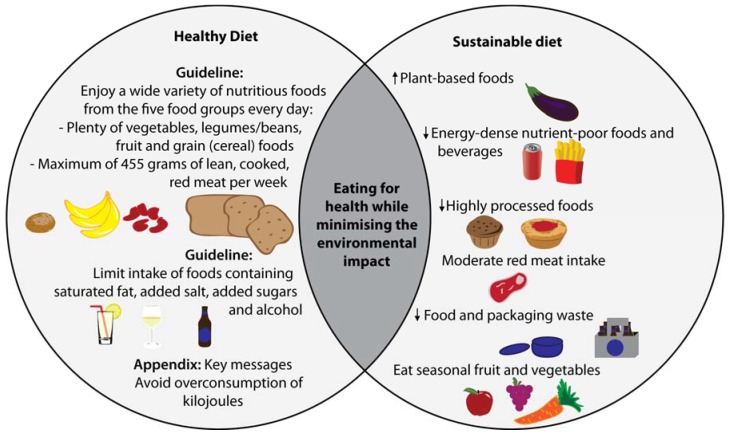
Graphic representation of the direct synergies between the 2013 Australian Dietary Guidelines and sustainable dietary behaviours outlined in the Australian Dietary Guidelines [7].

The Australian Dietary Guidelines, which provide the evidence-base for dietary recommendations and directions for nutrition policy in Australia, have highlighted the issue of food, nutrition and environmental sustainability over the last decade. The 2013 review of the Guidelines sought to assess the evidence to make dietary recommendations that were protective of health as well as the environment. However, no specific guidelines to address a sustainable diet were made as a result of inadequate evidence in the area, rather an appendix containing key messages regarding food, nutrition and environmental sustainability. These recommendations include advice to: try to eat seasonal produce; reduce food and packaging waste; and avoid overconsuming kilojoules [7]. Several of the Australian Dietary Guidelines form indirect synergies between eating a diet for good health and a sustainable diet to reduce burden on the environment (represented graphically in Figure 1). For example, the overconsumption of kilojoules is associated with overweight and obesity, but is also creating an avoidable environmental burden due to the resources used in the production, storage and preparation of food [7,15,16]. While attempts to create awareness of the impact of dietary choices on the environment exist, the absence of set guidelines relating to sustainable diets in Australia is the probable result of limited evidence in this area of nutrition and no dietary assessment method to accurately measure an individual’s healthy and sustainable dietary behaviours.

There is no agreed definition for what constitutes a “healthy and sustainable diet”. Separately, healthy diets conform to the Australian Dietary Guidelines [7], while sustainable diets have been defined by the Food and Agriculture Organization of the United Nations as “those diets with low environmental impacts which contribute to food and nutrition security and to healthy life for present and future generations. Sustainable diets are protective and respectful of biodiversity and ecosystems, culturally acceptable, accessible, economically fair and affordable; nutritionally adequate, safe and healthy; while optimizing natural and human resources.” [4]. Several European countries have developed guidelines for a healthy and sustainable diet [17,18] and research examining associations between other dietary recommendations and dietary patterns and their associations with environmental sustainability is becoming available [3,13]. Even with strengthening evidence on the health benefits of diets with lower environmental impact, the revised Australian Dietary Guidelines failed to include specific sustainable eating dietary recommendations [3,19].

There is a plethora of evidence suggesting climate change and poor health are two major public health concerns, both of which would benefit from government policy promoting more sustainable dietary behaviours [2,9,20,21]. However, dietary recommendations and policies cannot be developed without an evidence base. In order to collect evidence on how current dietary patterns adhere to a sustainable diet, surveillance and monitoring of individual dietary behaviours using a comprehensive dietary assessment method is required.

To the authors’ knowledge there is no feasible dietary assessment method to accurately measure an individual’s healthy and sustainable dietary behaviours and the need for such a method has been highlighted in a recent review by Johnston *et al.* [22]. To date methods of dietary assessment have focused mostly on nutrients and food groups and not considered the assessment of sustainable dietary practices, such as reducing food packaging and waste. Brief assessment instruments, commonly used in population surveillance, have been used to reliably estimate the quality of diets in Australia [23]. These methods typically use a short questionnaire or several questions to assess knowledge and specific diet and nutrition behaviours [24,25]. Other frequently used dietary assessment methods, such as written food records, provide more objective data on what individuals are eating and in some cases individuals may be asked to record food waste. However, a limitation of written food records is there is no way to verify the recording and researchers must rely on good literacy levels, the ability of people to accurately estimate portion sizes and remember to write down all meals, snacks and beverages, creating burden on participants. The use of technology in dietary assessment, and more specifically image-based food records, is a new and rapidly emerging area that will reduce the burden for participants through the elimination of detailed writing and portion size estimation. Image-based dietary assessment methods, including the mobile food record (mFR) application, enable people to capture their intake by taking a momentary image and do not allow users to review, edit or alter earlier images [26,27,28,29,30]. This feature may reduce the chances of people reflecting on their prior consumption and consequently underreporting further intake. In addition, before and after eating images taken using the mFR application allow for the assessment of plate waste and packaging use, as well as the estimation of serving and portion sizes. For such reasons the existing mFR application shows great potential as a feasible method for individual and population-wide nutrition monitoring of sustainable dietary behaviours. Food image data previously collected from a population-based sample of adults using the mFR will enable the validation of a *Healthy and Sustainable Diet Index.*

Diet quality indices assist in translating intake data collected using dietary assessment methods to values or scores that are more easily interpretable and allow for consistent comparisons between groups of interest. Such indices are developed to measure dietary patterns, behaviours and adherence to particular eating recommendations in populations [31]. Diet quality indices consider multiple components of a diet and apply weighting factors to each component to calculate a final diet quality score [32]. Developing and validating indices for use in dietary assessment can assist in guiding nutrition interventions, population monitoring, informing policy makers, monitoring the effectiveness of programs and research [33]. Examples of validated diet quality indices include the Healthy Eating Index [33], Mediterranean Diet Score [34], Diet Quality Index [35], Dietary Guideline Index [31], Dietary Quality Score [36], Australian Recommended Food Score [23] and Dietary Approaches to Stop Hypertension (DASH) Diet Score [37].

Traditionally, dietary indices have been developed to monitor specific or general nutrient intake and predict the effect of dietary behaviours on health outcomes. But, there is increasing need to measure impacts of dietary behaviours on external factors due to the potential negative impact on the future of the food supply (e.g., the environment) [22]. A recent review by Johnston and colleagues highlighted the urgent need to develop innovative approaches to measuring and promoting sustainable diets so consumers and policymakers can become aware of the benefits on individual and population health and the environment [22]. In doing so, the authors emphasised the need for culturally acceptable and locally appropriate indices to accurately assess sustainable diets, suggesting the development of such indices would enable the measurement of a suite of indicators relating to the impact of dietary behaviours on health and the food system to inform policy makers [22]. This paper addresses the gap in the literature by proposing a feasible method to assess multiple elements of a healthy and sustainable diet.

This paper describes the protocol and methodology for a proposed novel dietary assessment method to measure indicators of an individual’s healthy and sustainable diet not typically measured in traditional methods. Due to a lack of consensus of what constitutes a healthy and sustainable diet, the five dietary behaviours selected for assessment were chosen based on the evidence documented in the Australian Dietary Guidelines, and Appendix G on Environmental Sustainability [7]. The five characteristics of a healthy and sustainable diet selected relate to nutritional status and/or future food supplies to maintain good health. As the proposed dietary assessment method uses images to assess healthy and sustainable dietary behaviours, the selection has been confined to those that can be objectively assessed from food and beverage images using a mFR. The five indicators to be assessed using the mFR application include the intake of ultra-processed EDNP foods and beverages, individually packaged foods and beverages, fruit and vegetables (including seasonality), dairy, eggs and meat, and plate waste.

Food intake data, collected using the image-based mFR during the Connecting Health and Technology study, will provide evidence to assist the development of this method and a *Healthy and Sustainable Diet Index*, which will provide evidence for policy makers, health professionals, and others interested in promoting environmental sustainability through dietary recommendations (e.g., the agricultural sector). A validated index to accurately assess healthy and sustainable dietary behaviours, and ultimately gather evidence on individual eating behaviours, is timely and urgent. It is yet to be determined but the mFR may have the potential to be a cost effective method to gather valuable data on healthy and sustainable dietary behaviours, an area of nutrition in need of evidence. The dietary assessment method described in this paper when implemented will provide evidence on current adherence to a healthy and sustainable diet, addressing a gap in the literature both in Australia and globally. This protocol paper outlines the methods used to assess healthy and sustainable dietary behaviours using an mFR, providing detail to assist with further advancements in this field of dietary assessment and allowing for future reproducibility. Importantly, the methods proposed in this paper may address the lack of dietary assessment methods to assess sustainable dietary behaviours, as highlighted in the review by Johnston *et al.* [22].

## 2. Experimental Section

### 2.1. Study Participants

The study sample to be used for developing the proposed methods will consist of 247 adults aged from 18 to 30 years, comprising of 162 (66%) women and 85 (34%) men previously recruited for another study, the Connecting Healthy and Technology study, referred to as CHAT [26]. Recruitment involved sending letters of invitation to 15,000 residents from 57 suburbs (using the Socio-Economic Indexes for Areas) in the Perth Metropolitan Area from the Federal Electoral Roll, a compulsory enrolment system for Australians aged over 18 years. Participants were screened either online, using a survey website, or on the telephone to ensure the inclusion criteria were satisfied (aged between 18 and 30 years and owned a mobile phone). For the original study, potential participants were excluded if they were: (a) unable to attend on four occasions to complete the 6-month randomised controlled trial; (b) studied nutrition; (c) took part in extreme forms of exercise; (d) followed a restrictive diet; or (e) pregnant or breastfeeding. The CHAT study was conducted between July 2012 and June 2013.

The Connecting Health and Technology study was registered on the Australian and New Zealand Clinical Trials Registry (ACTRN12612000250831) and approved by the Curtin University Human Resources Ethics Committee (HR181/2011) and the Western Australian Department of Human Research Ethics Committee (#2011/90).

### 2.2. Study Design

During the Connecting Health and Technology study, participants completed four day mFRs using the CHAT application at baseline and at the end of the 6-month randomised controlled trial. During both visits height and weight data were collected. Participants were asked to capture before and after images of all eating occasions using a mobile device (iPod Touch) provided by the research team. On the initial visit participants were taught how to use the specifically designed dietary assessment method, the CHAT application, uploaded onto the iPod Touch (iOS6). Participants were asked to place a small fiducial marker in the bottom left hand corner of every image to assist in portion size and colour estimation [38]. Details of the mFR CHAT application have been previously described by Kerr *et al.* [39].

Participants were asked to take images over four consecutive days, from Wednesday to Saturday. On completion of the food record, participant’s clarified the contents of images with an Accredited Practising Dietitian, and verified plate waste or if the leftover food or beverage was consumed at a later stage. Each image obtained during the CHAT study contains metadata on the time, date and location it was taken, allowing for the assessment of whether fresh produce were in season at time of consumption. The food images collected during the CHAT study will be used to validate and test the *Healthy and Sustainable Diet Index*.

**Figure 2:**
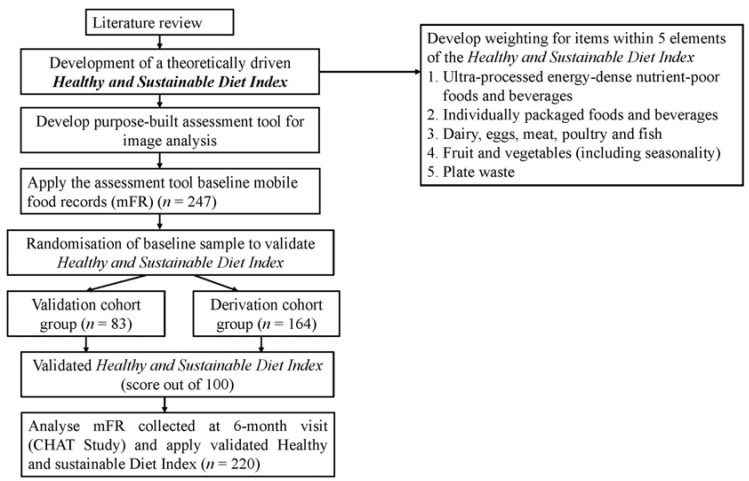
Flow chart of study design.

For the development of the proposed methodology, participants completed an mFR at baseline (*n =* 247) and repeated this six months later (*n =* 220). A secondary analysis by the Accredited Practising Dietitian who was involved in the original analysis of all food images collected during the CHAT study will take place. A purpose built Microsoft Access database will be developed to assess the contents of images, and the review of each image pair (including a before and after eating image). A minimum of one image pair per day will be required to be considered a valid day. Five indicators of a healthy and sustainable diet will be assessed using the images captured using the mFR. An objective of this study is to determine whether the five selected dietary behaviours can be assessed from image-based food records without interaction with participants. This is therefore a “proof of concept” approach to developing the Index before replication in possible future interventions. A flowchart outlining the design of the proposed study can be seen in Figure 2.

### 2.3. Assessment of Healthy and Sustainable Dietary Behaviours

For the development of a *Healthy and Sustainable Diet Index*, dietary behaviours were identified from evidence of their supportive or unsupportive role within a healthy and/or a sustainable diet and the inclusion of details that can be assessed using images. Descriptions of each component’s role within the context of a healthy and sustainable diet are outlined below. The five indicators to be assessed using the mFR application include: the intake of ultra-processed EDNP foods and beverages, individually packaged foods and beverages, fruit and vegetables (including seasonality), dairy, eggs and meat portions, and plate waste. These dietary behaviours will be assessed using the following proposed methodologies.

#### 2.3.1. Ultra-Processed EDNP Foods and Beverages

Processed foods form a large component of modern diets and have been linked to the growing rates of overweight and obesity [7,40,41,42]. A definition of food processing is “all methods and techniques used by industry to turn whole fresh foods into food products” [43]. Food processing is important to ensure an adequate and safe food supply [44], however, high levels of food processing often increases the energy density of food due to “ultra-processing” with the addition of added fat and sugar. In general, foods that have been highly or ultra-processed are more likely to contain high levels of saturated fats, added sugars and/or sodium and minimal levels of micronutrients therefore are often categorised as “energy-dense nutrient-poor” choices [41,45]. Energy-dense nutrient-poor foods and beverages are associated with poor diet quality, overweight, obesity and chronic disease [45,46], as well as being some of the most emissions-intensive food products due to the processing, packaging and landfill necessary to produce these foods in certain locations [9]. Although all EDNP foods may not negatively affect the environment more than other food items, these foods are generally low in nutrients [7]. In addition, the excessive intake of kilojoules above an individual’s energy requirements, from these EDNP foods and beverages, is a dietary behaviour unsupportive of health whilst creating unnecessarily burden on natural resources [7,15]. Highly processed takeaway foods and poor diet quality are associated with abdominal obesity in young adults [40,47] and compared to older age groups, young adults are more likely to consume EDNP that are convenient, highly processed and packaged, such as meat pies, fried potatoes, pizzas, crisps, confectionary, savoury pastries, chocolate and sugar-sweetened beverages [40,48]. Hence, processed food is not the issue in a healthy and sustainable diet, the issue is EDNP ultra-processed foods.

Ultra-processed foods are defined as those that require minimal, if not any, culinary preparation [41]. Previous studies have relied on household expenditure surveys and semi-quantitative food frequency questionnaires to assess the intake of ultra-processed foods [41,42,43], yet no studies have investigated the consumption of ultra-processed foods in Australia. A unique aspect of the methods to be used is that EDNP ultra-processed foods will be assessed using image-based mFRs. For inclusion in this component only ultra-processed foods and beverages categorised as energy-dense and nutrient-poor, such as cakes, crisps, commercial burgers and sugar-sweetened beverages will be used [7]. Ultra-processed EDNP foods and beverages will be assessed according to the Australian Dietary Guidelines serve sizes-one serve of EDNP food or beverage being equivalent to 600 kJ (143 kcal) [7]. Nutrient dense foods that are highly processed, such as bread, will be excluded due to associated health benefits.

**Figure 3:**
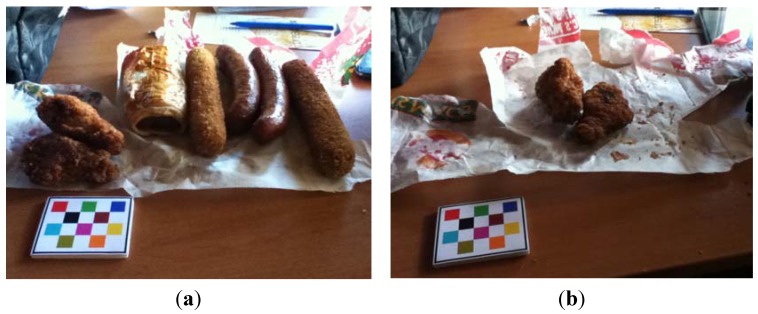
Example of using an mobile food record (mFR) to assess ultra-processed energy-dense nutrient-poor foods. (**a**) Before eating image; (**b**) After eating image shows the food waste. The number of energy-dense nutrient-poor food and beverage (EDNP) serves consumed were: 1 sausage roll (commercial, 175 g) = 2100 kJ, fried sausage (large) 1300 kJ × 2 = 2600 kJ, crumbed fried sausage (large) 1800 kJ × 2 = 3600 kJ → Total: 8300 kJ/600 kJ = 14 serves of EDNP foods.

An example image of ultra-processed EDNP foods collected using the mFR application can be seen in Figure 3. Using the proposed assessment protocol, this eating occasion would be recorded as 14 serves of ultra-processed EDNP foods. The two pieces of fried chicken appearing in the after eating image would not be counted as ultra-processed EDNP serves but rather non-compostable food waste.

We can appreciate all EDNP foods may not necessarily be worse for the environment than other food items, however, in regard to health consequences, these food items offer minimal, if any, nutritional benefit. In addition, the excessive intake of kilojoules above an individual’s energy requirements, from these EDNP foods and beverages, is a dietary behaviour unsupportive of health whilst creating unnecessarily burden on natural resources.

#### 2.3.2. Individually Packaged Foods and Beverages

Food packaging plays a crucial role in maintaining a safe food supply and has the ability to reduce waste by retaining the effect of food processing to extend shelf life [49]. However, food packaging negatively impacts the environment at a number of stages including during production, transport and land fill [15]. Individually packaged foods are convenient and are becoming more common in Australian supermarkets. An Australian study assessed attitudes towards environmentally friendly eating behaviours and found people believe food packaging has a greater impact on the environment compared to the consumption of meat [50].

Key messages in Appendix G of the Australian Dietary Guidelines encourage people to select foods with appropriate packaging and recycle due to the impact on natural resources [7]. Food packaging is not assessed by traditional dietary assessment methods. Images from mFRs show great potential for the accurate assessment of the intake of individually packaged products as they are easily identifiable from the before eating images. Due to the negative impact of packaging on the environment, all individually packaged items will be recorded regardless of the nutrient composition of the food it originally contained. However, individually packaged foods, classified as either EDNP or healthy, will be counted separately to allow for further assessment of adherence to a healthy and sustainable diet. Foods and beverages served from larger packages (so not individually packaged) such as a glass of milk poured from a two litre bottle will not be recorded due to the unavoidable use of larger packages to ensure a safe food supply. A limitation of this method is that some food or beverages may be removed from individual packaging prior to the before eating image being taken. This challenge could be avoided by requesting participants do not remove individual packaging prior to taking images using the mFR.

**Figure 4:**
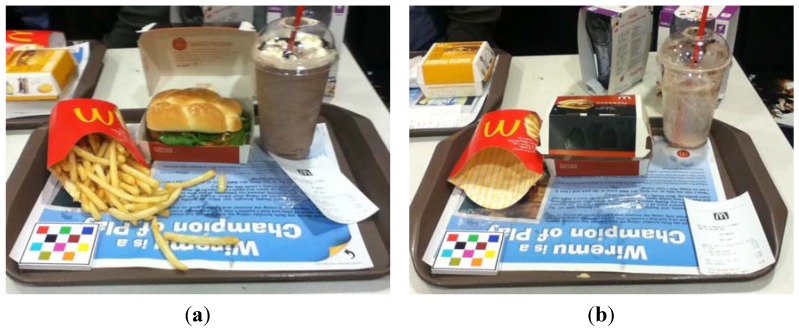
Example of using the mobile food record (mFR) to assess individually packaged foods. (**a**) Before eating image; (**b**) After eating image.

An example of an image containing individually packaged items collected using the mFR application can be seen in Figure 4. Using the proposed protocol, this eating occasion would likely be assessed as containing two individually packaged EDNP food items and one individually packaged EDNP beverage item.

#### 2.3.3. Fruit and Vegetables

A diet consistent with the Australian Dietary Guidelines can help maintain a healthy weight and assist in the prevention of chronic diseases, such as cardiovascular disease, type 2 diabetes and some cancers [7,8,20,51]. Previous studies have found Australian adults eat less than the recommended daily serves of fruit and vegetables [52], supported by the most recent Australian Health Survey which found only three per cent of young adults meet the recommended two 150 gram serves of fruit and five 75 gram serves of vegetables per day, compared to 9.6% of older adults [53]. The reasons why people are not eating enough fruit and vegetables are complex but household income and the expense of fruit and vegetables have been shown to be significant factors [54,55]. In recent years, changes in climate have influenced the availability and affordability of some fresh fruits and vegetables in Australia [22,56]. The cost of fresh fruit and vegetables in Australia appears to be increasing at a higher rate than other food categories, evidenced by an 18.8% increase in the cost of fresh fruit and 10.7% increase in fresh vegetables in Western Australia since 2010 [57].

Diets high in fruit and vegetables have a lesser impact on the environment than those high in processed foods or animal-based foods [3,15]. Although the consumption of a diet that consists of mostly fresh fruits and vegetables is encouraged [7], it has been suggested that additional considerations need to be included, for example, produce grown locally and in season. This is because fruits and vegetables grown locally or in season are less likely to require a climate controlled environment and typically undergo less processing, packaging, transportation and storage [58,59]. However, other studies have suggested the benefits of consuming locally grown seasonal produce is not the determining factor of environmental impact because food production has more impact on the environment than transportation [12,60,61]. A recent study by Drewnowski *et al.* [11] assessed the relationship between nutrient and energy density and carbon footprint, and found that processed and frozen fruit and vegetables had a low carbon footprint when considered as per 100 grams in comparison to meat and dairy products. But when looking at energy density per 100 kcal, the carbon footprint of frozen and processed fruit and vegetables increased dramatically [11]. This study pointed out that carbon footprint is only one of many metrics to assess the environmental impact of food. Overall, it is widely accepted that some fruits and vegetables are more emissions-intensive than others depending on several factors, including country of origin, the need for protected conditions, storage and cooking.

Choosing seasonal local fruits and vegetables requires specific knowledge of where food was grown. This information is not always evident or available to consumers [7] particularly when meals are prepared by others (e.g., meals eaten at a restaurant). Studies have shown people are prepared to buy local produce, although factors such as convenience, price, accessibility and perceived quality also determine purchasing habits [50,62].

Using the protocol outlined in this paper, the intake of fruit and vegetables will be analysed from each eating image pair and classified according to the Australian Dietary Guidelines serve sizes (1 serve of vegetables = 75 grams, ½ cup cooked vegetables or 1 cup of salad vegetables, and 1 serve of fruit = 150 grams, 1 medium piece or 2 small pieces of fruit). A Microsoft Access database tool will be created to record the estimated serve size and type of fruit and vegetable consumed separately. This feature will allow for further assessment of the environmental impact of different varieties. As the time and date stamp is available from images collected with the mFR, the date fruits and vegetables were consumed can be recorded and merged within the database containing information on when each fruit and vegetable is in season. For example, in Western Australia bananas are in season in summer and autumn, meaning if someone consumed a banana between 1st December and the 30th May then the banana will be recorded as “eaten in season”. For this study, seasonal fruits and vegetables will be classified according to the Western Australia (WA) Seasonal Fruit and Vegetable Calendar [63].

Dietary assessment of seasonal and local fruit and vegetable intake poses significant challenges including: the additional burden of recording “place of origin” at time of purchase; prepared food not carrying this information (e.g., buying a salad at a café): and the sale of fruits and vegetables all year around, regardless of seasonality. A limitation of assessing the intake of seasonal fruit and vegetable intake is that there is no way of determining the origin of fresh produce, for example a banana from Queensland could be eaten in Western Australia, 4341 kilometres away by road (the main transportation method used). However, consuming seasonally available produce, regardless of origin, is likely to reflect an aspect of a healthy and sustainable diet. There is currently limited data on the intake of seasonal fruits and vegetables by adults in Australia. As less than 7% of Australians consume the recommended daily serves of vegetables and less than half consume the recommended two serves of fruit [64], increasing intake alone, regardless of seasonality, would result in health benefits.

**Figure 5:**
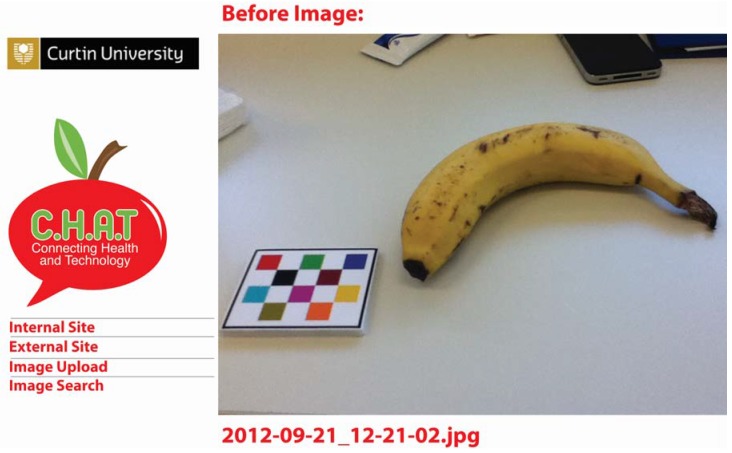
Example of using the mobile food record to assess seasonal fruit and vegetable intake.

An example of fruit to be consumed can be seen in the mFR image (as it appears using the web application hosted on a secure server) in Figure 5. Using this example containing the date of the image, one serve of fruit (e.g., one medium banana) would be entered in the database and because this eating occasion took place in September in Western Australia, this piece of fruit would be considered eaten “out of season”.

#### 2.3.4. Dairy, Eggs and Meat Products

There is substantial evidence supporting the additional environmental impact of meat and dairy foods, compared to plant-based foods [2,14,65]. Along with a growing population and urbanisation, there is a global transition from largely plant-based diets to diets higher in EDNP foods and animal-based foods [45], increasing burden on the food system. While the consumption of dairy foods in Australia is generally below dietary recommended levels, Australians traditionally consume large volumes of meat, with the consumption of beef constituting the highest amount [15]. Meat and dairy products from ruminant cattle and sheep are some of the greatest greenhouse gas contributors in modern diets [8,9].

Previous research in the area of sustainable diets has highlighted a healthy and sustainable diet can be followed without the complete exclusion of dairy and meat [1,2,8,65], however, excessive red meat and processed meat consumption has been linked to an increased risk of colorectal cancer [7,66]. To accommodate this, the latest review of the Australian Dietary Guidelines reduced the standard serving size of lean red meat to a set 65 grams from the previous range of 65 to 100 grams of cooked meat, with a maximum of seven serves, or 455 grams, of red meat per week [7]. In Australia, only 2.1% of people avoid red meat [53]. Therefore, comparing meat intake between small, moderate and large meat consumers is relevant in assessing a healthy and sustainable diet when only a small percentage of the population are vegetarians [67].

When applying the proposed method to this component, food images will be used to estimate average daily intake of milk, cheese and yoghurt, eggs and meat products (including red meat, poultry and fish). The volume of specific types of dairy, eggs and meat products (e.g., beef mince) will be recorded as an approximate gram or millilitre weight and compared to the Australian Dietary Guidelines recommendations [7]. Meat consumed in other food products assessed using this method, such as a beef patty in a commercial burger, will be counted as ultra-processed EDNP food serves and also meat serves.

#### 2.3.5. Food Waste

Australians throw away $5.3 billion Australian dollars (AUD) worth of food each year. This includes fresh food (AUD $2.9 billion), frozen food (AUD $241 million), take-away food (AUD $630 million), unfinished drinks (AUD $596 million) and leftover food (AUD $876 million) [68]. Young adults waste more food than older adults with 38% of 18–24 year olds wasting more than AUD $30 on fresh produce per fortnight, compared to only seven percent of older adults [68]. Reducing food waste from production to consumption will decrease burden on the food system, in turn benefiting the environment [69]. Discrepancies were detected in an Australian study comparing reported household fresh food waste (AUD $4.6 million) and actual fresh food waste (AUD $8 billion), collected during a household garbage bin audit [68]. The methods proposed here will accurately capture an important element of food waste, consumer plate waste, through the use of before and after eating food images, to support an area of research with a lack of sufficient data [69].

Image pairs allow for the accurate assessment of plate waste due to the presence of before eating and after eating images. Plate waste will be estimated as a percentage of food or beverage not consumed in the after eating image. Food waste in each image will also be classified as compostable (e.g., fruit, vegetables, egg shells), not compostable (e.g., meat and dairy) or unable to determine.

**Figure 6:**
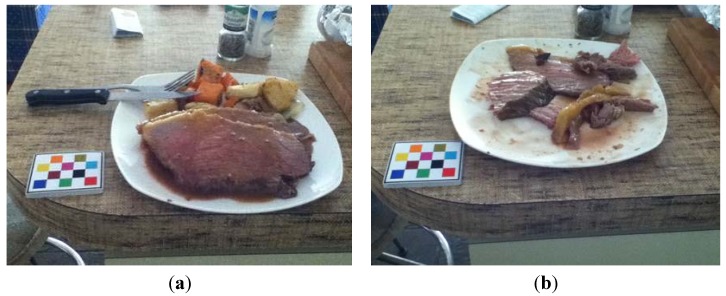
Example of using the mobile food record (mFR) to assess food waste and meat intake. (**a**) Before eating image; (**b**) After eating image.

An example of red meat intake and food waste can be seen in Figure 6. Using the described protocol, this eating occasion will be assessed as four serves of roast beef, and having 30% edible plate waste. Note, one serve of cooked beef is 65 grams [7].

### 2.4. Outcome Variables

The outcome variables measured using the proposed dietary assessment method include:

- number of serves and types of fruit and vegetables and whether they were in season at the time and location of consumption,
- intake of ultra-processed energy-dense nutrient-poor foods and beverages, separated by type and number of serves,
- intake of foods and beverages that are individually packaged, separated into “healthy” or “EDNP” foods or beverages,
- portion sizes and total amount of dairy, eggs and meat products
- percentage of plate waste and whether the food wasted was compostable.

### 2.5. Development of Healthy and Sustainable Diet Index

Existing diet quality indices will be reviewed to investigate the processes undertaken in development, validation, and evaluation and will guide the development of the *Healthy and Sustainable Diet Index*. This will be a theoretically driven Index, which will be internally validated using food image data collected during the CHAT study. Each indicator incorporated in the Index will be categorised into one or more of the following elements; impact on human health and/or impact on the environment. For example, ultra-processed EDNP foods and beverages impact health (contributing excess kilojoules and contributing to chronic disease risk) and the environment (use of water, electricity, transport and packaging). Another example is food waste, which has a direct negative impact on the environment (landfill) and a potential influence on health as fresh fruit and vegetables are perishable and often thrown away, creating a barrier for purchase and consumption.

The influence of dietary behaviours on human health will be given the highest weighting, followed by impact on the environment, for example plate waste. Weighting of different food items will involve a thorough assessment of available evidence, including evidence on the life cycle assessment of particular foods (which takes into account green-house gas emissions), and additional effects on ecosystems and biodiversity. The final Index may need to be modified when applied in other countries to take into account differences in the environmental impact of foods produced in various areas and climates, including climate conditions, farming, agricultural and production methods. For example the environmental impact of fruit that requires a climate controlled environment, *versus* seasonal fruit grown outside. A maximum number of total points will be allocated to each component of the *Healthy and Sustainable Diet Index*. A high weighting will not be given to components of the Index that cannot be measured accurately using image-based food records, for example whether fruits and vegetables consumed were locally grown and in season, due to the amount of error.

Each component incorporated into the *Healthy and Sustainable Diet Index*, will be given a weighting used to calculate a final score measuring adherence to a healthy and sustainable diet. For example, typically indices have a maximum score of 100, with a higher score indicating greater adherence to the preferred dietary pattern [23]. Individual components will be given a weighting to reflect consistency with the recommended dietary and sustainable eating outcomes, for example, fruit and vegetable intake may be given a higher weighting as it contributes to both healthy and sustainable eating.

The theoretically driven *Healthy and Sustainable Diet Index* will be internally validated for reliability, content validity and construct validity using 4-days image-based food records collected during a 6-month randomised controlled trial. The baseline data collected during the CHAT study (*n =* 247) will be randomised into thirds using age- and sex-stratified random sample techniques. Two-thirds of the sample will be randomly selected as the derivation cohort and the image-based mFRs of those selected will be used to develop the *Healthy and Sustainable Diet Index* using regression techniques. The remaining one-third of the sample will be used as the validation cohort for the *Healthy and Sustainable Diet Index*. This Index will also be used in the assessment of food images from the 6-month follow up (*n =* 220), and the results compared with baseline.

To assess content validity, components of the *Healthy and Sustainable Diet* *Index* will be assessed against the Australian Dietary Guidelines [7]. Construct validity will quantitatively assess how well the Index measures conformance to a healthy and sustainable diet.

To determine a total score using the *Healthy and Sustainable Diet Index*, density scores for each component will be calculated. Internal consistency, one form of reliability, will be assessed using Cronbach’s coefficient α. This test has previously been used in the evaluation of the Healthy Eating Index [70] to examine the degree of association between components, to determine if a diet only has one dimension. The relationship between the components of the *Healthy and Sustainable Diet Index* will be assessed using Pearson correlations coefficient. Principle component analysis will be used to assess if there are independent components of the Index. This will measure if there are any significant independent predictors of an overall score.

## 3. Discussion

The proposed methodologies described in this paper aim to determine if the mobile food record can be used to accurately measure five key indicators of a healthy and sustainable diet. The availability of dietary intake data collected from 4-day mFRs during the CHAT study enables the refinement of the assessment tool and internal validation of a theoretically driven *Healthy and Sustainable Diet Index*. This Index will be tested on a duplicate sample and a longitudinal sample of adults’ 4-day image-based food records to measure content validity, construct validity and reliability.

The *Healthy and Sustainable Diet Index* will be unique in two ways; firstly it will combine the assessment of eating behaviours that influence health outcomes (e.g., EDNP foods and beverages) and dietary behaviours that significantly burden the environment (e.g., ultra-processed foods, food waste). Secondly, it will require the use of image-based food records, which will enable the accurate assessment of dietary behaviours not assessed in traditional forms of dietary assessment (e.g., individually packaged foods).

Using an mFR application to assess the five healthy and sustainable dietary behaviours described in this paper has the potential for further enhancement of the mFR applications capability as a new dietary assessment method. For example, a current fiducial marker probe exists to alert users when the fiducial marker is not located in the image, as described by Ahmad *et al.* [29]. A similar mechanism to ask the user whether food waste detected in the after image was thrown in the rubbish bin, composted, saved for consumption at a later time or other could be incorporated into the mFR application.

Currently there is limited evidence on whether Australian adults have dietary habits consistent with a sustainable diet. Without adequate evidence in this area, appropriate changes to dietary recommendations and nutrition policy are challenging. Results from the Australian Health Survey indicate most Australian’s have eating habits inconsistent with the Dietary Guidelines, contributing to the burden of diet-related diseases in this country [63]. However, to the authors’ knowledge, there is currently no dietary assessment tool or indexing system to assess and monitor whether individuals have dietary behaviours inconsistent with a sustainable diet, such as the use of individual food packaging and plate waste.

Similar to other dietary assessment methods, using the mobile food record to assess dietary behaviours does not come without limitations. The primary limitation being if a participant forgets to take an image of an eating occasion. This can be minimized by the ability to set alerts on the mobile device to remind participants to take images of all foods and beverages consumed.

Although this method of dietary assessment was tested on a population-based sample of young adults during the CHAT study, the mFR has also been tested in other ages groups [71]. A unique aspect of this proposed work is that images collected using the mFR application have not previously been used to measure these important and topical dietary behaviours, and hold potential for accurate dietary assessment. In addition to the development and validation of a novel dietary assessment method, findings from the work proposed will provide evidence on the current healthy and sustainable dietary habits of young Australian adults.

## 4. Conclusions

The strengths of the protocol and methodology proposed include the development of a dietary assessment method to accurately assess key indicators of a healthy and sustainable diet that are not measured during traditional dietary assessment methods. This innovative method will enable the development of a *Healthy and Sustainable Diet Index* to assess an individual’s adherence to these dietary behaviours. The use of the mFR to assess adherence to a healthy and sustainable diet is a novel and innovative approach to dietary assessment. The steps outlined in this paper only capitalise on the images captured with the mFR, however other features in mobile devices, such as activity measures, could also be considered. Future applications of this method may strengthen this area of research, influence behaviour and raise the awareness of the potential benefits on individual and population health and the environment.
